# Letter from the Editor in Chief

**DOI:** 10.19102/icrm.2021.121207

**Published:** 2021-12-15

**Authors:** Moussa Mansour


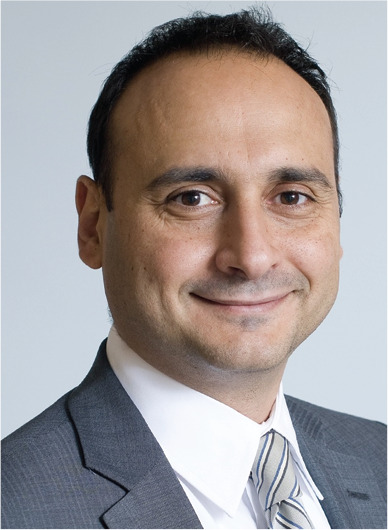


Dear Readers,

As 2021 concludes, I would like to summarize the major developments that occurred in the field of cardiac electrophysiology during the past 12 months. Overall, the field continued to grow at impressive rates, fueled by scientific and technical discoveries that helped to expand the indications and improve the outcomes of arrhythmia treatment.

First, interest in atrial fibrillation (AF) ablation continued to increase in 2021, and the rate of growth in the number of AF ablation procedures remains high. Strategies to improve the efficiency of the procedure, such as same-day discharge, have been adopted more broadly, allowing hospitals to continue performing AF ablations despite a nationwide shortage of hospital beds. On the device-manufacturing side, irreversible electroporation (also known as pulsed-field ablation) seems to be gaining significant momentum. This novel energy source for AF ablation has the unique feature of being cardio-selective, thus reducing and possibly eliminating the risk of injury to collateral structures,^1^ such as the esophagus and the phrenic nerve, and it is expected that this option will replace thermal energy in a couple of years.

The field of left atrial appendage closure (LAAC) also experienced significant growth in 2021. New-generation devices, such as the WATCHMAN FLX (Boston Scientific, Marlborough, MA, USA) and the newly approved Amulet™ device (Abbott, Chicago, IL, USA), appear to facilitate procedural success and reduce complications^2^ and will likely increase the accessibility of LAAC for a larger number of patients and providers. Large multicenter clinical trials, such as CHAMPION-AF (NCT04394546) and the Amplatzer™ Amulet™ Left Atrial Appendage Occlusion vs. Novel Oral Anticoagulation (CATALYST) trial (NCT04226547), were also launched in mid-2021 with the aim of seeking new indications for LAAC.

Ablation for ventricular tachycardia continues to gather significant interest. The procedure complication rate continues to decline because of new practices and studies, such as the Direct Oral Anticoagulants for Stroke Prevention Post–Ventricular Tachycardia Ablation (STROKE-VT) study, which demonstrated that the use of direct oral inhibitors following endocardial and/or epicardial ablation was associated with a reduced risk of transient ischemic attack or stroke.^3^

Finally, the field of cardiac implantable electronic devices has also witnessed exciting developments. The use of subcutaneous implantable cardioverter-defibrillators continues to increase, driven by the results of the PRAETORIAN clinical trial published in 2020.^4^ On the pacing front, left bundle branch area pacing is also gaining more popularity; this year, the Geisinger–Rush Conduction System Pacing Registry showed that, compared to conventional right ventricular pacing, this form of pacing was associated with a significant reduction in the composite outcome of all-cause mortality, heart failure hospitalization, or upgrade to biventricular pacing.^5^

I believe that we are fortunate to be working in a field witnessing growth on so many fronts. I would like to end by wishing you and your loved ones a happy holiday season.

Sincerely,


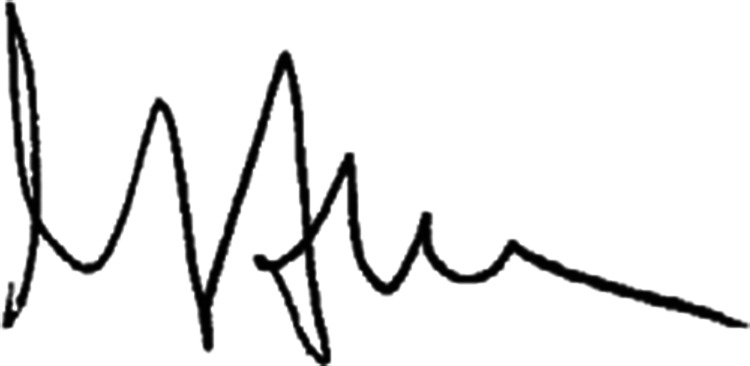


Moussa Mansour, md, fhrs, facc

Editor in Chief

*The Journal of Innovations in Cardiac Rhythm Management*

MMansour@InnovationsInCRM.com

Director, Atrial Fibrillation Program

Jeremy Ruskin and Dan Starks Endowed Chair in Cardiology

Massachusetts General Hospital

Boston, MA 02114
